# The epidemiology and medical care costs of *Echinococcus granulosusis* in Jahrom, southern Iran from 2007 to 2017

**DOI:** 10.1080/20008686.2020.1821503

**Published:** 2020-09-24

**Authors:** Hadi Khatonaki, Samaneh Mazaherifar, Gholamreza Shokoohi, Naser Hatami, Zahra Vafai, Farshid Javdani, Ahmad Abolghazi

**Affiliations:** Department of Medical Parasitology, School of Medicine, Jahrom University of Medical Sciences, Jahrom, Iran

**Keywords:** Cystic echinococcosis, epidemiology, Jahrom, Iran

## Abstract

**Background:** Echinococcus granulosus is a rare parasitic infection causing Cystic Echinococcosis, which can be dangerous due to involving the body. This parasitic infection is a significant health problem in Iran. However, little is known about this disease, specifically in Jahrom city; thus, we aimed to investigate the epidemiology and the economic impact of the illness.

**Methods:** In this descriptive cross-sectional study, the files of 137 patients who were under the care, and treatment of the final diagnosis of Cystic Echinococcosis were evaluated by reviewing the information such as age, gender, occupation, place of residence was collected, and analyzed.

**Results:** Human cystic echinococcosis cases were more common in females, 57.2% (12 patients) and 42.8% (9 patients) were male. In terms of age, most patients (23.8%) were in the age range from 21 to 30 years. The chief complaint at diagnosis, in all cases, was abdominal pain. Besides, 71.42% of the cases had the liver involvement alone, 9.52% had the lung involvement alone, 9.52% had a co-infection of liver and lung, and 4.74% had the kidney involvement alone.

**Conclusions:** The results of the present study are beneficial in determining the disease status and the epidemiology of hydatid cyst in this area.

## Introduction

**Figure 1.**
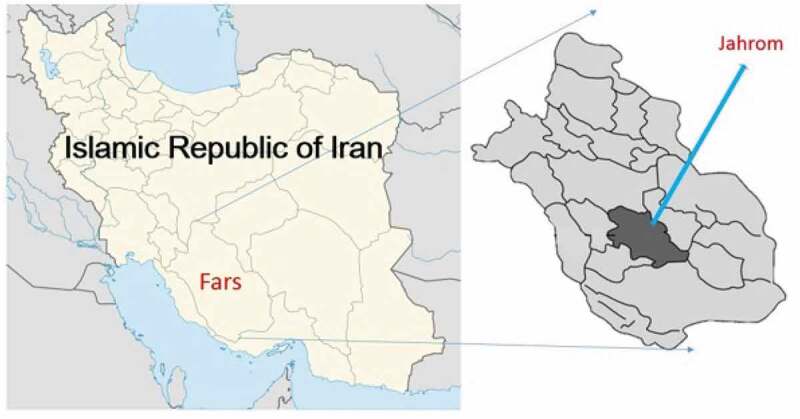
The geographical situation of the area of study, Jahrom, Fars, Iran.

*Echinococcus granulosus* is a worm of the cestode species. The larval stage of this worm causes a hydatid cyst, which is one of the most dangerous parasitic diseases affecting humans or domestic animals [1]. This cyst consists of several layers, including the laminated sheet, the Germinal layer,and the brood capsule containing protoscolices [2]. The parasite is transmitted by swallowing eggs of worms in water and foods and having contact with infected animals such as dogs. Hydatid cyst can form various parts of the human body, including the liver, lungs, brain, and heart, and cause disease [3]. The disease has been reported from different countries of the world, especially the eastern ones. Iran is among the countries with the highest prevalence of parasites, such as *Echinococcus granulosus* [4,5]. The annual mortality rate for *Echinococcus granulosus* is between 2 and 4%. Various methods are utilized for the diagnosis of the disease, e.g., radiography, CT scan, and ultrasound. The gold standard for the treatment is the surgery, in addition to using drugs such as mebendazole and albendazole [6]. The tearing of cyst involves a high risk of its compressive effect on adjacent organs and tissues, which can lead to sudden death [7]. Due to the risk of hydatid cyst and its lethal side effects in humans, control of the disease agent among domestic animals, dogs, and humans is essential. Various epidemiological studies have been carried out to identify these high-risk areas and perform prevention in those areas. In Iran, there have been many studies on the epidemiological study of this parasite in livestock. Its animal prevalence in dogs has been reported to be 33% as the primary host of the disease in Iran [8–10]. Considering that less attention has been paid to these studies in Jahrom city (Fars province, Iran), we reviewed the epidemiology of this disease in this southern Iranian city from 2007 to 2017.

## Materials and methods

Study area: Jahrom is one of the neighboring cities of Fars province, located in the southern part of the region. The town is 5436 km^2^ in area and borders Shiraz in the north to Shiraz city, east to Kerman province, south to Hormozgan province, and west to Bushehr province. The city is located within longitudes of 52° 45′ to 54° 4′ E and latitudes of 28° 19′ to 29° 10′ N. The population of the city in 2017 had 228,532 inhabitants. Jahrom city has two university hospitals: Peymanieh and Motahari. (Figure 1) The current study is a descriptive retrospective epidemiological study that reviews the patient referrals to health centers in Jahrom city from 2007 to 2017. The patients in this study were the patients who had been diagnosed with a hydatid cyst in a hospital. Data extracted from patients’ records were extracted including age, gender, marital status, duration of hospitalization, initial diagnosis, final diagnosis, the complaint at referral, job, place of birth, and diagnostic method. The statistical analyses on the collected data were performed using SPSS ver. 21. The following formula calculated the incidence rate [11].

$$Incidence\;Rate=\frac{New\;cases}{Population\;at\;risk}\times 10^{N}\left[11\right]$$

Patients were contacted if their data needed to be completed with patients by phone call. Ethical permission(s): this study approved by Jahrom University of Medical Sciences Id: IR.JUMS.REC.1396.005

## Results

**Figure 2.**
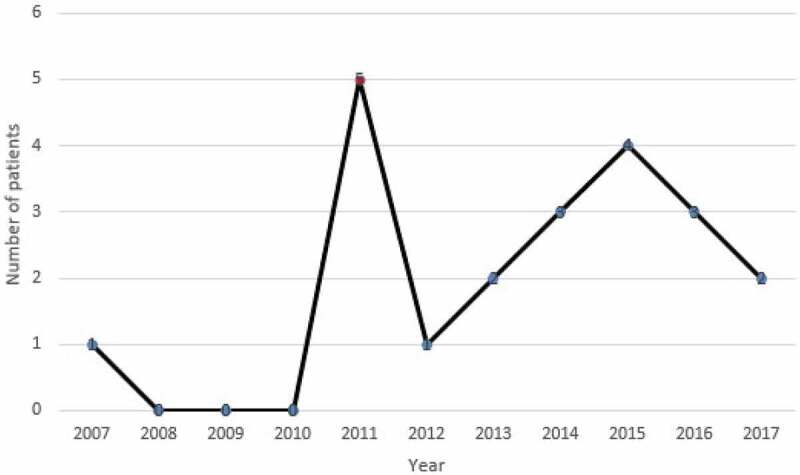
Human incidence Cystic Echinococcosis cases in Jahrom’s health centers between 2007 and 2017.

**Figure 3.**
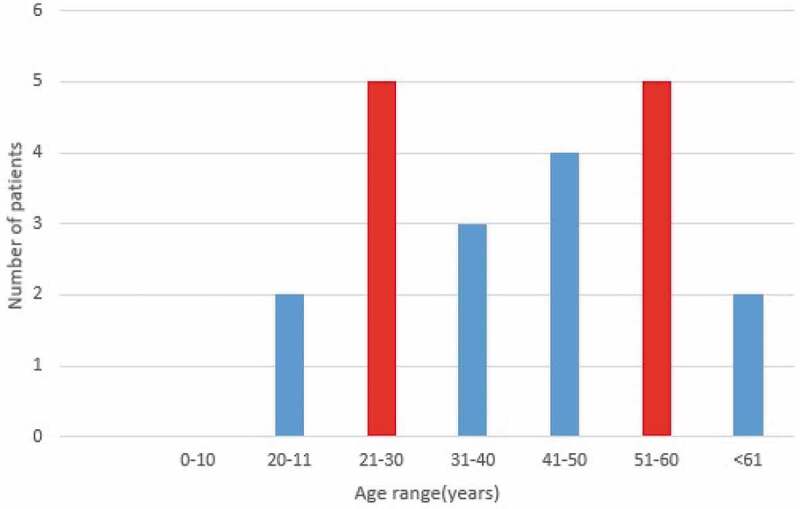
The age range of the infected patients with Cystic Echinococcosis in Jahrom’s health centers between 2007 and 2017.

**Figure 4.**
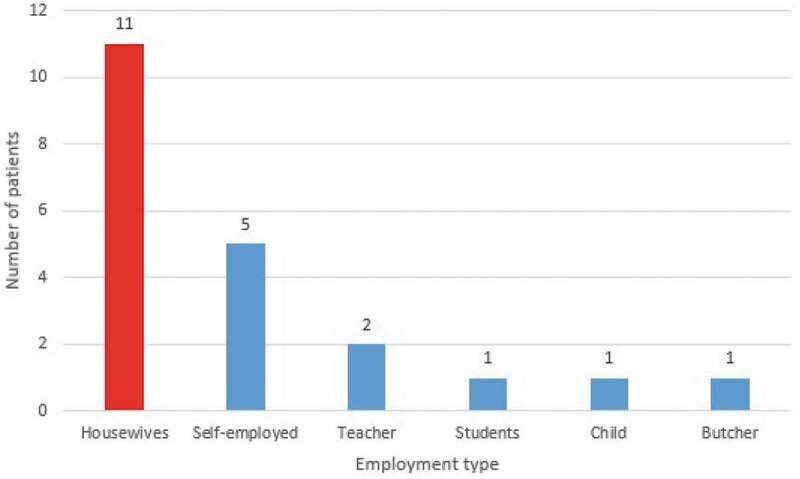
The job of the infected patients with Cystic Echinococcosis in Jahrom’s health centers between 2007 and 2017.

**Figure 5.**
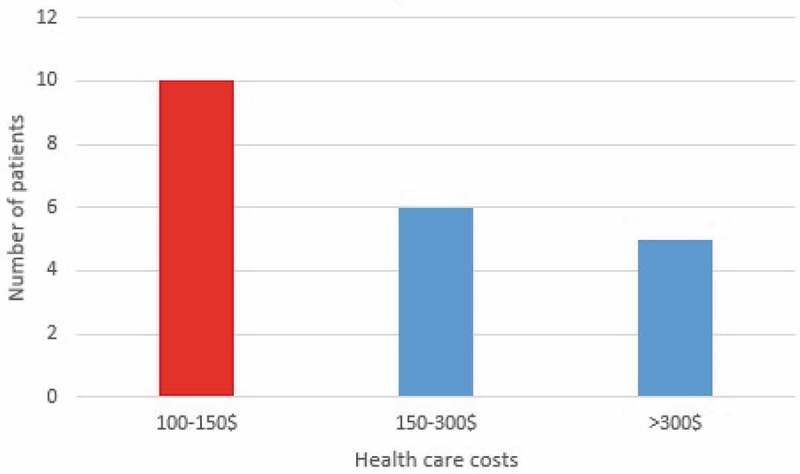
The health care costs imposed on infected patients with Cystic Echinococcosis in Jahrom’s health centers between 2007 and 2017.

**Figure 6.**
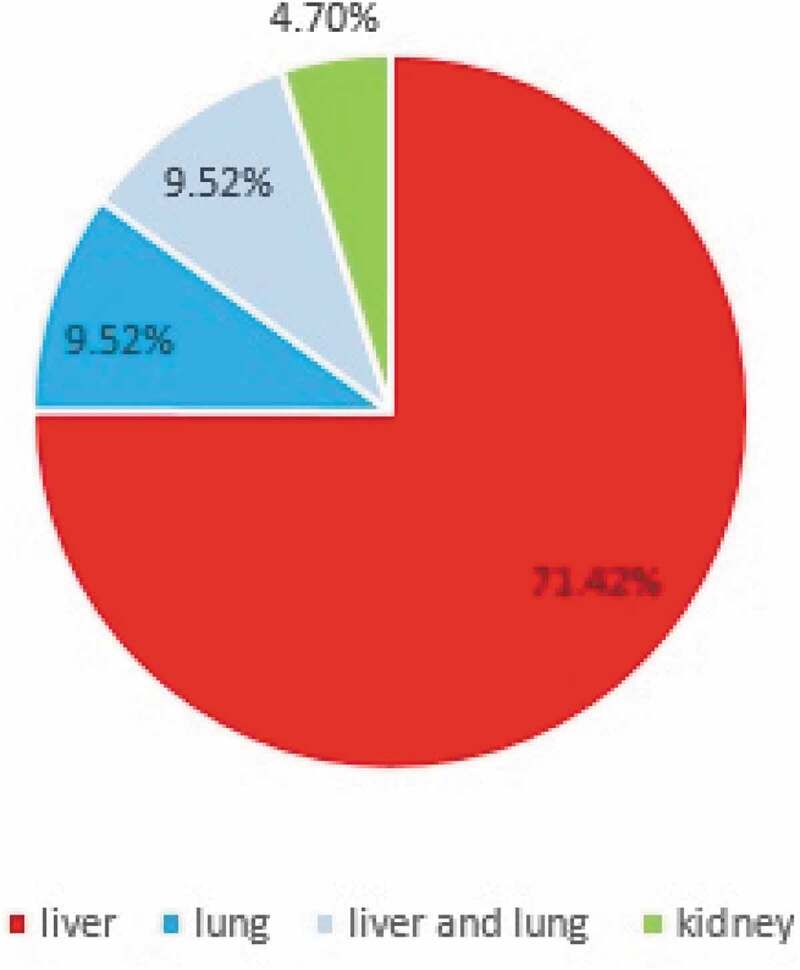
Localization of hydatid cyst in patients from Jahrom’s health centers between 2007 and 2017.

From 2007 to 2017, 21 cases of hydatid cyst were registered in patients referred to Jahrom city hospitals. (Figure 2). Among these cases, 57.2% (12 patients) were women, and 42.8% (9 patients) were male. Also, 38.9% (8 patients) were urban residents, and 61.91% (13 patients) were rural residents. In terms of age, most patients (23.8%) were in the age range from 21 to 30 years and 51 to 60. About 9.52% of cases were under the age of 20 years. (Figure 3). According to the obtained results, 28.24% of the patients were from the surrounding area of Jahrom, and the rest were residents of Jahrom city and its suburban areas. In terms of job, 52.38% were housewives, 23.80% were self-employed, 9.52% were teachers, 4.76% were students, 4.76% was children, and 4.76% was a butcher. (Figure 4) The cost of admission to the hospital and the operation of these patients is displayed in (Figure 5). In total, the economic burden on the target community was 4600 USD (estimated total cost in 21 patients). In terms of clinical symptoms, the chief complaint at diagnosis, in all cases, referring to the Jahrom Hospital, was abdominal pain. Also, cough and fever were seen in 19.04% and 9.52% of them, respectively. Besides, Localization of hydatid cyst in patients is shown in. (Figure 6).

## Discussion

Iran is one of the countries where *Echinococcus granulosus* infection is endemic [12]. From 2007 to 2017, a total of 21 hydatid cyst patients was hospitalized in Jahrom, a city in the southwest of Iran. Compared to the community of Tehran (the capital city of Iran), which is 8.5 million in the latest census, the population of Jahrom is much lower (about 200,000). In a study conducted by Shoaei et al. on the epidemiology of hydatid cyst in Tehran, this health issue was reported in 81 patients over a decade [12]. At the same time, in Jahrom, with a population of about one-forty of Tehran’s society, we observed 21 cases of this disease. This result demonstrates a relatively high incidence of hydatid cyst in Jahrom. Given the fact that this city is near to the provincial capital city (Shiraz) with more top health facilities, and given the fact that patients are more likely to go to this provincial capital, the number of people who are affected by this disease may be higher than the reported rate. The annual incidence of hydatid cyst in Jahrom was reported to be 1.05 cases per 100,000 people, while it was 1.33 per 100,000 people in Hamedan province, southwestern Iran [13], and 3 cases per 100,000 people in Kashan [14].

In this study, a somewhat higher percentage of women than men were infected. The women were pimarily housewives (11 women are housewives, and one woman is a teacher). This result is consistent with the findings of Mehrabani et al. in the northern part of Iran and Parkouhi et al. in East Azerbaijan [15,16]. In the present study, the percentage of patients with hydatid cyst living in the villages was more than that of the residents of the city, which is in line with the study of Ahmadi et al., who carried out an identical study. Haji Pirlo et al. presented similar results for Hamedan province in the northwest of Iran and East Azerbaijan [13,16,17]

According to Parkouhi et al. and Ahmadi et al., the prevalence of hydatid cyst in people living in urban areas was higher in Northern Iran [13,15]. In the present study, the highest frequency of involvement was in the age group of 26–35 years, which is consistent with a study by Abdul Hamid et al. in Iraq [18].

Meanwhile, in a study by Parkouhi et al. in northern Iran, the highest age range involved was 51–60 years [15]. In this study, the chief complaint of patients was abdominal pain, which is in accordance with the review by Schantz et al. [19]

The highest percentage of hydatid cysts was found in the liver with 71.42% and then the lungs with 9.52%. In a study by Abdul Hamid et al. in Iraq, the most involved body organs with this cyst were liver and then lung, while in the study of Parkhuhi et al., the most significant involvement was in the liver and then the spleen [16–19]. The average medical cost on the studied population was 288.83, USD which was matched by a study by Rokni et al. [20].

## Conclusion

Cystic Echinococcosis is an essential neglected disease; in this study we found a relatively high prevalence in the city of Jahrom in Iran. Therefore considering this disease is important to be able to prevent it. Accurate determination of risk factors and treatment costs are useful for formulating health policies for the prevention and treatment of hydatid cysts.
